# HARE: Supporting Efficient Uplink Multi-Hop Communications in Self-Organizing LPWANs

**DOI:** 10.3390/s18010115

**Published:** 2018-01-03

**Authors:** Toni Adame Vázquez, Sergio Barrachina-Muñoz, Boris Bellalta, Albert Bel

**Affiliations:** Department of Information and Communication Technologies, Universitat Pompeu Fabra, Carrer de Roc Boronat 138, 08018 Barcelona, Spain; sergio.barrachina@upf.edu (S.B-M.); boris.bellalta@upf.edu (B.B.); albert.bel@upf.edu (A.B.)

**Keywords:** LPWAN, IoT, M2M, multi-hop, power saving mechanisms

## Abstract

The emergence of low-power wide area networks (LPWANs) as a new agent in the Internet of Things (IoT) will result in the incorporation into the digital world of low-automated processes from a wide variety of sectors. The single-hop conception of typical LPWAN deployments, though simple and robust, overlooks the self-organization capabilities of network devices, suffers from lack of scalability in crowded scenarios, and pays little attention to energy consumption. Aimed to take the most out of devices’ capabilities, the HARE protocol stack is proposed in this paper as a new LPWAN technology flexible enough to adopt uplink multi-hop communications when proving energetically more efficient. In this way, results from a real testbed show energy savings of up to 15% when using a multi-hop approach while keeping the same network reliability. System’s self-organizing capability and resilience have been also validated after performing numerous iterations of the association mechanism and deliberately switching off network devices.

## 1. Introduction

In the coming years, electronic equipment will be interconnected and consequently almost every person and industry will become simultaneously data generators and consumers. The Internet of Things (IoT) paradigm is a key enabler of this vision by delivering machine-to-machine (M2M) and machine-to-person communications on a massive scale.

As more and more things are connected to the Internet, low-cost and low-traffic devices are starting to be demanded. However, traditional cellular networks do not deliver a good combination of technical features and operational cost for those IoT applications that need wide-area coverage combined with relatively low bandwidth, long battery lifetime, low hardware and operating cost, and high connection density [1].

**Figure 1:**
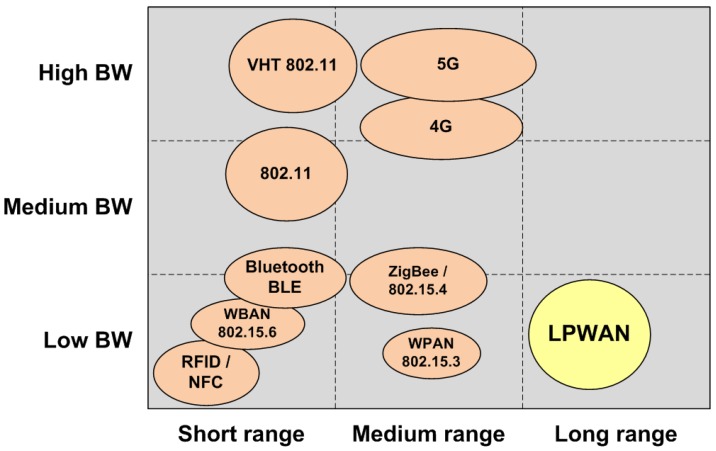
Localization of LPWAN technologies according to range capability and bandwidth required.

Low-power wide area networks (LPWANs) are intended to become the engine of long-range, low-bandwidth IoT applications (see Figure 1), which until now have been constrained by deployment costs and power issues. The goal of these networks is to deliver small amounts of data over long ranges, at rates of up to tens of kilobits per second (kbps), with a battery lifetime of up to several years, supporting thousands of devices connected to a base station, and facilitating online integration.

LPWAN architecture is characteristically single-hop, where end devices are connected directly to the base station by following a star topology, greatly simplifying the network and endowing it with robustness and centralized control. However, this single-hop massive channel access sets out some inherent challenges: reliability, scalability, flexibility, and quality of service (QoS). In fact, the channel access mechanism of some LPWAN technologies resorts to the use of ALOHA [2,3], a random medium access control (MAC) protocol in which end devices transmit without doing any carrier sensing to check the channel state in advance. Although simple, this uncontrolled medium access leads to interference or packet collisions among uncoordinated devices, acutely affecting reliability and scalability in dense networks [4,5,6,7,8,9]. In addition, LPWAN devices located far away from the base station must make use of high transmission power levels, resulting in severe energy consumption and reduced battery lifetime [10].

In this article, the HARE protocol stack is proposed as a new LPWAN technology flexible enough to adopt uplink multi-hop communications when proving energetically more efficient than single-hop. A full set of advanced techniques belonging to different communication layers has been designed for this purpose, while ensuring data transmission reliability:

- Network synchronization, with nodes being periodically set in time by means of beacons emitted from the base station.
- Time division multiple access (TDMA)-like channel access for groups of contenders with multiple transmission opportunities.
- Adaptive transmission power level.
- Flexible and scalable network association process.
- Energy-aware, adaptive and resilient routing protocol.
- Regular use of deep-sleep states.

A HARE protocol stack has been implemented and tested in real hardware platforms. Results evaluation from different network configurations (single-hop vs. multi-hop, use of different radio duty cycling protocols, channel error injection) shows very high reliability while maintaining low energy consumption (particularly in multi-hop topologies). Furthermore, a better overall system’s behavior when using multi-hop topologies in non error-prone scenarios has been observed.

The remainder of this paper is organized as follows: Section 2 presents the current state-of-the-art in LPWANs and their multi-hop approaches. Section 3 introduces the main requirements of feasible use cases for HARE. Next, Section 4 describes the general operation of the protocol stack and Section 5 provides detailed information of the developed mechanisms. Section 6 describes the system’s implementation in three testbeds and Section 7 compiles the obtained results from different experiments. Lastly, Section 8 presents the conclusions and discusses open challenges.

## 2. State-of-the-Art

There are increasingly more LPWAN competing technologies, each employing varied techniques to achieve long range and low power operation [11]. This section reviews the most representative examples of the two main types: *licensed* and *unlicensed spectrum LPWANs*, and surveys their currently existing multi-hop approaches.

### 2.1. Licensed Spectrum LPWANs

*Licensed spectrum LPWANs* are deployed in the spectrum holdings of mobile operators and take advantage of cellular technologies to expand into new IoT usage scenarios, by providing improved support for low-cost and energy-efficient machine type communication (MTC) devices. NarrowBand IoT (NB-IoT), enhanced machine-type communication (eMTC) (also known as LTE-M), and extended coverage GSM IoT (EC-GSM-IoT) are the initiatives emerging from the 3rd Generation Partnership Project (3GPP) Release 13 [12].

### 2.2. Unlicensed Spectrum LPWANs

*Unlicensed spectrum LPWANs* use any of the publicly available open spectrums in the industrial, scientific and medical (ISM) bands and can be in turn classified into three categories:

1. Proprietary systems: SIGFOX™ [13], Ingenu™ [14], and Telensa [15].
2. Systems under an alliance: LoRa™ [16], Weightless™ [17], DASH7 [18], and Wi-SUN [19].
3. Standards: IEEE 802.11ah (also known as Wi-Fi HaLow) [20], ETSI low throughput networks (ETSI-LTN) [21], and Wireless M-BUS (EN 13757-4) [22].

### 2.3. Multi-Hop Approaches in LPWANs

Most LPWAN technologies operate in the sub-1GHz band to achieve large coverage ranges, and are built following a star topology, where end devices are directly connected to the base station. Though this single-hop approach simplifies the network design, it makes communications rely heavily on transceivers’ capabilities (i.e., transmission power, antenna gain, data rate, etc.). In contrast, in low- or medium-range networks, usually operating at 2.4 GHz, IoT-aimed multi-hop environments are common:

- IETF RPL (IPv6 routing protocol for low-power and lossy networks) [23] is a distance vector routing protocol, which provides a mechanism whereby multipoint-to-point traffic from devices inside a low power and lossy networks (LLN) towards a central control point, and vice versa.
- Time-slotted channel hopping (TSCH) is one of the MAC modes defined in the IEEE 802.15.4e standard [24]. It combines time-slotted access and channel hopping, thus providing predictable latency, energy efficiency, communication reliability, and high network capacity. TSCH is topology independent, as it can be used to form any network topology (e.g., star, tree, partial mesh, or full mesh) [25].
- A new Working Group called 6TiSCH was created at the IETF to enable IPv6 over the TSCH mode of the IEEE 802.15.4e standard. In addition, 6TiSCH manages the schedule created by the time-slotted access of TSCH and matches it against the traffic needs in the network. Its distributed scheduling mode allows nodes to agree on a schedule by using distributed multi-hop protocols and neighbor-to-neighbor negotiation [26].

From combining both conceptions (long range links and heterogeneous network topologies) in the aforementioned unlicensed spectrum LPWANs, novel multi-hop approaches have been proposed in recent times:

- LoRaBlink [27] is a protocol on top of LoRa’s physical layer designed to support reliable and energy efficient multi-hop communications. Time synchronization is used to define slotted channel access, with a reliability of 80% according to tests. While downlink messages are distributed through flooding, nodes use a directed flooding approach for uplink communications.
- One of the main singularities of DASH7 is enabling both star and tree topologies to facilitate the management of large networks [28]. In the latter case, stations (STAs) not directly reaching the gateway (GW) may transmit to sub-controllers or other STAs, which forward the messages to the GW. Regarding the MAC layer, STAs check the channel periodically for possible pull requests or downlink transmissions.
- To improve data transmission efficiency and extend range coverage, IEEE 802.11ah allows for using a dual-hop relay system in between the AP and end stations [29]. According to simulations from [30], these benefits are especially important for uplink communications. In addition, since an STA is closer to the relay than to the AP, it can transmit frames (i) at lower power and (ii) at higher data rate making the transmission shorter, consequently reducing its energy consumption.
- To reduce the time an STA is competing for the channel as well as to increase its sleep time, IEEE 802.11ah uses a scheme called *TIM and Page Segmentation* [31], which has inspired the hybrid MAC sublayer of HARE. Thus, distribution of STAs into groups is used not only for organizational purposes but also for effectively allocating available channel resources.

Unlike the aforementioned multi-hop approaches, HARE considers energy consumption as a key element in the decision making processes of the different network self-organization mechanisms (for instance, routing or power management). The typical single-hop approach of LPWAN technologies is therefore called into question and even replaced by multi-hop topologies when proving energetically more efficient. Furthermore, HARE’s high flexibility when allocating channel resources ensures communications reliability, network scalability, and facilitates future adoption of QoS schemes.

## 3. Scenarios and Requirements

According to their own characteristic range and bandwidth capabilities with respect to other technologies, the main use cases to which LPWANs are addressed include security alarms, car park spaces, agricultural applications, smart metering, consumer electronics, and intelligent buildings. By way of illustration, in the ENTOMATIC EU-project [32] a network of wireless sensor nodes [33] periodically reports information on pest population density and environmental parameters, such as temperature and relative humidity.

HARE is clearly aligned with typical LPWAN applications and circumscribes its suitability to those scenarios with special concern for energy efficiency, where device batteries are so limited that the establishment over time of a direct connection to the base station, or gateway, would greatly affect their lifetime.

In this sense, Table 1 offers a comprehensive list of common requirements from use cases to which HARE protocol stack gives response in combination with the appropriate hardware. Assuming that this hardware provides good signal penetration, a single GW could serve up to thousand low-traffic devices within its given coverage range. Applications executed by STAs, in turn, follow a *continuous data delivery model* [34] for their sensed information, periodically delivering small amounts of non-delay sensitive data. Although not considered in the current article, future HARE developments will consider offering QoS in scenarios with miscellaneous sensors (continuous, event-driven, query-driven, and hybrid).

As sensor nodes are scattered over large areas, sometimes with problematic access, self-maintenance of the system shall be a priority, capable of giving response to the following challenges:

1. Node initiated network connection: once installed for the first time or relocated, any node shall initiate its association process through a simple action (for instance, pressing a button).
2. Self-configuration and management: with the aim of building a robust network, it shall adapt itself to environmental and/or topology changes without human intervention.
3. Battery lifetime maximization: LPWANs replace old monitoring systems consisting of assigning human resources to study in situ the behavior of one or more physical parameters. Therefore, maximizing battery lifetime in such systems is vital in order to justify their usage ahead of other methods.
4. Firmware distribution: any change in the network configuration or in the application purpose shall be remotely and easily distributed by the GW.

Lastly, although the operating system of embedded sensor nodes is typically less complex than general-purpose operating systems [35], it shall cope with the high variety of resources to manage in this kind of devices (processors, memories, clocks, network interfaces, and so forth) and the demand of support for concurrent execution of processes (time synchronization, data acquisition, task scheduling, channel access, routing parameters, and so forth).

Under these premises, Table 2 compiles some of the main use cases supported by the HARE protocol stack in five IoT representative sectors: home and industrial automation, public infrastructure, natural resources, and smart agriculture and farming.

## 4. HARE Operation

The HARE protocol stack conceives end devices as elements controlled by the GW by means of beacons. This centralized approach allows STAs to remain asleep the majority of the time, so that their single concern is to be awake enough in advance to listen to the next beacon. Network synchronization is thus achieved and allows the GW to ask for specific data and/or distribute configuration changes easily.

The GW is considered to be appropriately placed close to a power source or an energy harvesting solution. Therefore, it may always stay in an active state and is provided with the ability to directly communicate (i.e., via single-hop communications) with any node of the network through unicast and/or broadcast messages as well as to redirect gathered data to other networks or the Internet.

**Figure 2:**
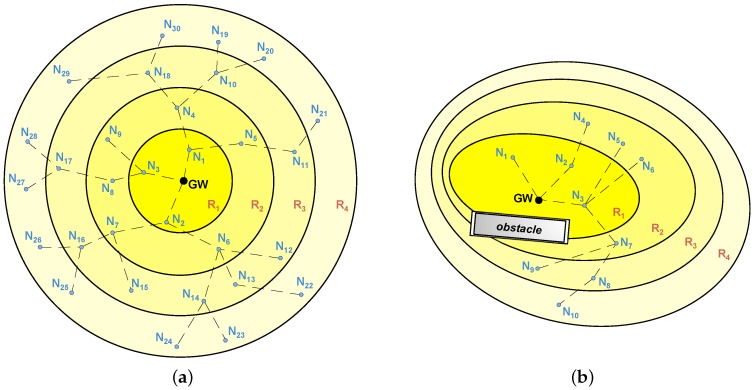
Network topology of typical multi-hop LPWANs. (**a**) multi-hop LPWAN consisting of a GW and 30 STAs ($N_{1}$–$N_{30}$) deployed in four rings ($R_{1}$–$R_{4}$); (**b**) multi-hop LPWAN affected by an obstacle, with a GW and 10 STAs ($N_{1}$–$N_{10}$) deployed in four rings ($R_{1}$–$R_{4}$).

Conversely, STAs can take advantage of their neighbors to create multi-hop paths over which data is transmitted to the GW by means of lower transmission power levels. During the association process, and depending on their position within these paths, STAs are ideally organized into *rings*, resulting in network topologies as the ones shown in Figure 2. The number of hops to reach the GW determines the ring number (i.e., STAs from ring 2 require two hops to reach the GW).

Each uplink data transmission phase (consisting of one or more *transmission windows*) begins with a beacon signal from the GW. Transmission windows are in turn virtually split into as many TDMA slots as network rings, so that STAs are only active during their own slot (for transmitting data) and the previous one belonging to their children (for receiving data). *Children* refers here to all STAs of an adjacent higher ring from which an STA receives packets. Similarly, *parent* refers to that STA from an adjacent lower ring to which an STA transmits its own packets (after aggregating the ones from its children) in its way to the GW.

The first slot is allocated to the highest ring and the rest are scheduled consecutively. Data received by STAs is aggregated to that generated by themselves, and finally sent to the corresponding parent at the minimum power level that ensures reliable communications. This process is repeated as many times as rings that the network has.

The correct reception of data transmissions at the GW is acknowledged at the end of every transmission window with a broadcast message, so that STAs are not only aware of their own end-to-end reliability, but also of those STAs in the same path to the GW. These acknowledgment beacons, together with the information obtained from their adjacent nodes, allow STAs to decide whether they should remain awake to perform retransmissions of lost network packets in successive transmission windows.

Network association (also started by a beacon) remains stable until a change in the topology is detected or the mechanism is reset by the GW. Nevertheless, the agreed transmission power between adjacent nodes in the association phase is constantly monitored and adjusted in a decentralized way in order to reduce the energy consumption.

## 5. Protocol Stack

The main features of the HARE protocol stack are shown in Table 3 and a complete description of its different layers is offered next.

### 5.1. PHY Layer

HARE protocol stack is intended to be used over any wireless PHY layer fulfilling a minimum set of functions; namely, availability of different operational states both in the microprocessor (processing, low power mode) and in the radio module (receiving, transmitting, and sleeping), selection of different transmission levels in the radio transceiver, and ability to execute low level tasks required by typical shared medium access techniques.

### 5.2. Link Layer

**Figure 3:**
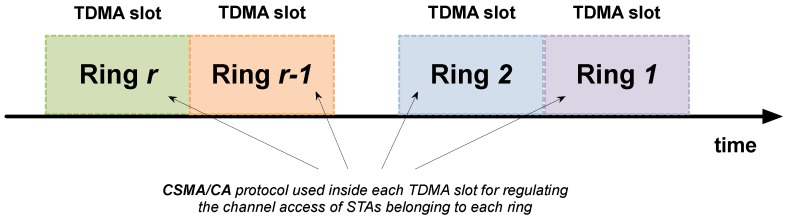
MAC sublayer consisting of TDMA slots and CSMA/CA technique inside them.

Link layer is split into the MAC sublayer and the radio duty cycling (RDC) sublayer (see Table 4). The MAC sublayer consists of two levels: (1) a TDMA scheme to divide the time in slots; and (2) a carrier sense multiple access with collision avoidance (CSMA/CA) technique performed by the group of STAs allocated in each slot (see Figure 3). Slots’ duration and STA allocation are managed by the GW. The RDC sublayer tries to keep the radio module turned off while providing enough rendezvous points for two nodes to be able to communicate with each other.

The use of a scheduling-based TDMA scheme in the MAC sublayer of HARE is mandatory to structure transmissions in ring-based slots. Conversely, both the contention-based protocol of the MAC sublayer and the RDC sublayer used in these slots can be freely chosen from state-of-the-art available options [36].

#### 5.2.1. Beaconing System

**Figure 4:**
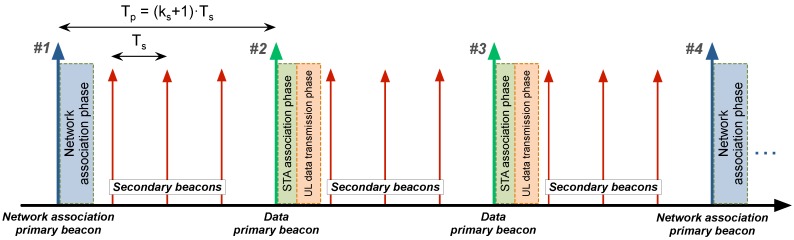
HARE beaconing system consisting of *network association primary beacons*, *data primary beacons*, and *secondary beacons*.

The designed beaconing system has a double function: synchronizing the network devices and scheduling the different actions to be performed in a time-division multiplexing scheme. Two types of beacons are used for this purpose: *primary* and *secondary beacons* (see Figure 4).

Both beacons include a timestamp, the time until the next *primary beacon*, and the next action to be taken by the network: for instance, an association phase (*network association primary beacon*), or an uplink data transmission phase (*data primary beacon*). *Secondary beacons* include the same information as *primary* ones, and are used to guarantee information redundancy for already associated STAs as well as to accelerate network discovery for non-associated ones. However, no action is performed by STAs after a *secondary beacon*.

Time between two consecutive *primary beacons* and two consecutive *secondary beacons* is defined as $T_{p}$ and $T_{s}$, respectively, where $T_{p}=\left(k_{s}+1\right)\cdot T_{s}$, with $k_{s}$ being the number of *secondary beacons* transmitted after every *primary beacon*.

#### 5.2.2. Wakeup Patterns

A wakeup pattern is a set of instructions generated by the GW that define the wakeup plan of its associated STAs over time periods. With the goal of minimizing the time STAs remain active (and, consequently, their energy consumption), two different wakeup patterns controlled by the GW are proposed according to the network’s traffic flow [37].

**Figure 5:**
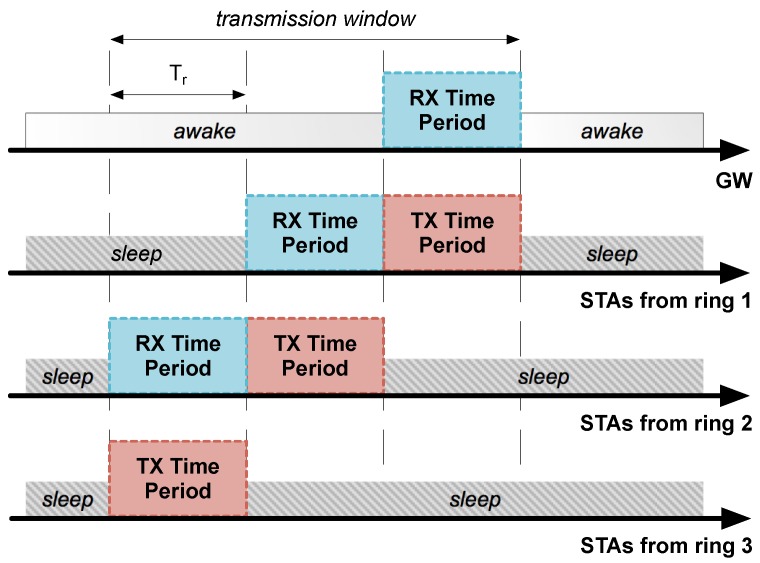
Example of a *staggered wakeup pattern* in a three-ring LPWAN with a single transmission window performing uplink communications.

The *periodic wakeup pattern* is suitable for listening to broadcast downlink communications from the GW, as it makes all STAs wake up at the same time. On the other hand, uplink communications follow a *staggered wakeup pattern*, which allocates different active periods (also called *ring slots*, with $T_{r}$ duration) to nodes belonging to adjacent rings with partial overlapping (as shown in Figure 5). Apart from reducing time STAs are awake during uplink communications, this method facilitates the implementation of data aggregation mechanisms.

Even though STAs have predetermined active periods, they can go to sleep even earlier in the transmitting (TX) time period if their parent has acknowledged all their data, or in the receiving (RX) time period after having received all data from their children.

#### 5.2.3. Data Transmission, Aggregation, and Segmentation

Downlink communications are generally executed through broadcast messages from the GW. Conversely, uplink communications are unicast and follow a multi-hop route. The *staggered wakeup pattern* fits here perfectly with the approach of data aggregation in WSN. Thus, nodes attach their own data to that received from their children and all the information is jointly sent to the next hop (i.e., parent). If the total amount of data aggregated by an STA exceeds the maximum payload supported by the hardware, it is split into segments sent consecutively. The amount of data aggregated by an STA (from itself and from its children) is called *packet*. If this packet is split into different parts, each one of these parts is called *segment*. In case both terms can be indistinctly used, the current article will use *packet*.

A selective ACK mechanism has been developed, so that, before the end of the allocated time slot, the receiver explicitly lists which segments in a stream coming from the same child are acknowledged. Upper layers are therefore responsible for making the sender retransmit only the missing segments in successive transmission windows.

#### 5.2.4. Power Regulation Mechanism (PRM)

The selection of the minimum suitable transmission power level for outgoing packets is managed through a mechanism based on the received signal strength indicator (RSSI). For this purpose, a safety margin for reliable communications is defined by ${\mathrm{RSSI}}_{\min}$ and ${\mathrm{RSSI}}_{\max}$. If a node is transmitting data packets (or ACKs) to its parent (or child) at a power level making the received RSSI higher than ${\mathrm{RSSI}}_{\max}$, it will be asked to decrease it for the next transmission. Similarly, if the received RSSI is lower than ${\mathrm{RSSI}}_{\min}$, it will be asked to increase it.

PRM requests are included in an RSSI control field of data packets and ACK headers. Possible values of this field are: *increase*, *keep*, and *decrease*. Once computed the requests from parent and children, the STA determines whether and how to regulate its own power level depending on the following considerations:

- If one or more STAs ask for a higher value, increase the power level.
- If all STAs ask for a reduction, decrease the power level.
- Otherwise, keep the current power level.

In addition, if an STA needs to retransmit a packet to its parent, it will also increase the power level in each new transmission window. Regarding the association process, whenever an STA listens to a discovery request, it will answer at maximum power. The STA selected as parent will keep the maximum power level at the beginning and regulate it by following the previously described procedure. Instead, those STAs not selected as parents will set their power back to the level they had before answering to the discovery request.

In summary, the main advantages of using such a link layer are:

- Clock synchronization is inherent to TDMA, with nodes being periodically set in time by means of beacons.
- Groups of nodes have their time slots clearly allocated, and collisions within groups are sensibly reduced or even avoided by using CSMA/CA.
- Network overall lifetime is increased by putting nodes in non-active modes for most of the time and only periodically waking up to check for activity.
- Association and routing mechanisms included in HARE also fit for this scheme, so that intermediate and already associated nodes do not have to constantly listen to hypothetical network discovery requests.
- The uplink transmission scheme is suitable for performing data aggregation methods.
- Changes in the network configuration or even new firmware can be easily distributed in a coordinated manner.

### 5.3. Network Layer

Network communications follow a centralized scheme, where the GW adopts the main role and assumes the responsibility of managing network associations, delivering network addresses, and periodically notifying the start of new routing processes.

STAs adopt a subordinate role waiting for orders coming from the GW. In the routing process, they organize themselves in paths autonomously, but all subsequent data transmissions are addressed to the GW, directly or through other STAs. Conversely, the GW can make use of its greater transmission power to periodically send broadcast messages to all network STAs, or send unicast messages to selected STAs.

#### 5.3.1. Addressing System

The addressing system is managed by the GW, which allocates a unique network address to each node during the association process. Nodes will maintain the same network address as long as they do not leave the network. A dynamic record matching the MAC and the network address of all STAs is stored in the GW. The size of the network address is configurable and its value determines the addressing range.

#### 5.3.2. Association

To cope with multiple association requests in a short period of time, the system is able to admit new STAs through two different mechanisms: an active, global, scheduled one, called *network association mechanism*; and a passive, singular one, called *STA association mechanism*.

- **Network association mechanism**
  The *network association mechanism* allows a large amount of STAs to associate to the network in a short period of time. Once the GW is activated, or after a pre-determined number of *primary beacons* $\left(N_{\mathrm{pr}}\right)$, the GW broadcasts a *network association primary beacon*.
  Depending on the RSSI value received in the *network association primary beacon* and three configuration parameters embedded in it (maximum RSSI, number of association turns, and amplitude in dB of each turn), STAs determine their association turn (generally, the greater the RSSI received, the earlier association turn is selected). Then, a number is selected uniformly at random by the STA, determining its association slot within the association turn. The number of association turns $\left(a_{t}\right)$, association slots per turn $\left(a_{s}\right)$, and the length of a slot $\left(T_{a}\right)$ are parameters set by the GW and included in every *network association primary beacon*.
  STAs then follow with a discovery message sent via broadcast, which is responded by the GW and all the already associated STAs, provided they are within the coverage range. The process of selecting the best path to reach the GW is detailed in Subsection 5.3.3. After taking into account all the received association requests during a $T_{g}$ time period, the GW notifies the joining of new STAs by means of a summary broadcast message sent immediately after every association turn.
- **STA association mechanism**
  The *STA association mechanism* provides a solution to those specific nodes that (i) have not found a path to the GW during the *network association mechanism*; (ii) have been powered on between two consecutive *network association primary beacons*; or (iii) have simply suffered routing problems in their path to the GW.
  This mechanism follows the same pattern as the *network association* one, with the single exception that there is only one association turn located immediately after each *data primary beacon* to be used by non-associated STAs.

Inactive or erratic STAs are removed from the network and the GW’s routing table to create, if necessary, new routing paths that ensure correct packet reception from remaining network STAs. Disassociations can be controlled by the GW through the *disassociation mechanism* or by the STAs themselves through the *self-disassociation mechanism*:

- **Disassociation mechanism**
  The GW removes an STA from the network if not receiving any data packets during a pre-determined number of consecutive *primary beacons* $\left(N_{\mathrm{pd}}\right)$. A roster with the latest disassociated STAs is included in every *primary beacon*. This information is not only useful for malfunctioning STAs, which can make immediate use of the *STA association mechanism*, but also for their parents, as they can check the current state of their children. Hence, if all its children became disassociated, a parent would go to sleep during the RX time period allocated to its ring.
- **Self-disassociation mechanism**
  The goal of this mechanism is to avoid repetitive association requests and other energy-consuming procedures that could make STAs run out of battery when no connection with the GW is possible. All STAs have a timer that is activated after being switched on or when receiving a *primary beacon*. From that moment on, if an STA does not receive any other beacon during a predetermined period $\left(T_{d}\right)$, it turns itself off. Thus, the STA is considered *dead* and it will need to be reactivated by manual procedures.

#### 5.3.3. Routing

HARE uses its own distance vector routing protocol inspired by RPL to build a destination oriented directed acyclic graph (DODAG) [23]. This protocol is only executed as part of the *Network association mechanism* and the *STA association mechanism*, so that there is no continuous routing packet exchange among neighbors. Thus, according to the responses to the discovery message coming from other nodes, each STA determines the best candidate to become its parent in its path to the GW; i.e., the node with the minimum *S* value from:

(1) $$S=a_{1}\cdot \left(P_{{\mathrm{TX}}_{max}}-{\mathrm{RSSI}}_{\mathrm{TX}}\right)+a_{2}\cdot \left(P_{{\mathrm{TX}}_{max}}-{\mathrm{RSSI}}_{\mathrm{RX}}\right)+a_{3}\cdot r+a_{4}\cdot c,$$

where $P_{{\mathrm{TX}}_{max}}$ is the maximum transmission power of the transceiver (in dBm), ${\mathrm{RSSI}}_{\mathrm{TX}}$ is the RSSI received at the candidate (in dBm), ${\mathrm{RSSI}}_{\mathrm{RX}}$ is the RSSI received at the STA itself (in dBm), *r* is the ring to which the candidate belongs, and *c* is the current number of candidate’s children. The *a* weights are attached to every *primary beacon*, and can be tuned by the GW according to environmental requirements (A study on selection of optimal routing configuration parameters in multi-hop LPWANs like HARE can be found in [38].).

Once the best parent is computed, the STA sends it a unicast request. This request will be forwarded by the parent through its own path until reaching the GW, which will send a packet via broadcast confirming the association and providing the requesting STA with its new address. This way, both the newly associated STA and its parent are informed of the establishment of the new path.

When the association process is finished, the STA exactly knows which is its ring, and the next hop its messages must follow to reach the GW. As long as the STA is associated to the network, it uses the same routing path, which is only recomputed after an internal or external (i.e., from its parent) failure. Indeed, no new routing process is initiated unless it is part of a new *network association* instance.

### 5.4. Transport Layer

Reliable end-to-end communications from network STAs to the GW, where retransmissions are only executed when needed and by the minimum number of involved devices, are achieved in HARE by using the mechanisms described below.

#### 5.4.1. End-to-End ACK

**Figure 6:**
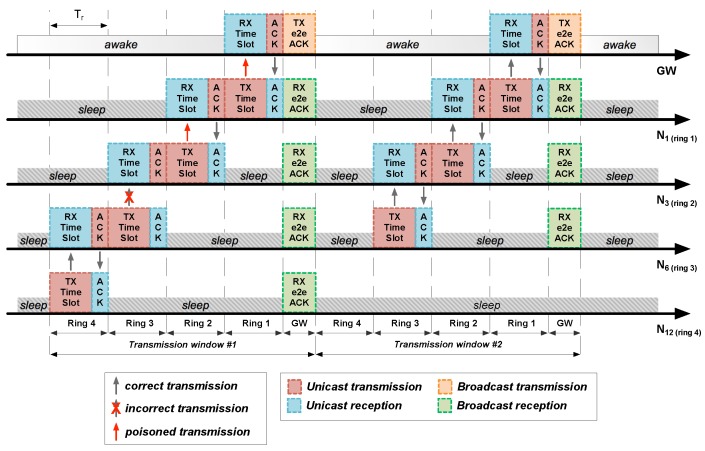
Uplink data transmission phase in a multi-hop LPWAN running HARE protocol stack with two transmission windows and the network topology from Figure 7. Note the communication problems in the first transmission window between nodes $N_{6}$ and $N_{3}$.

According to the *staggered wakeup pattern*, STAs from ring 1 are the last ones to access to the channel and transmit their information. Once compared the data sources with the expected uplink traffic, the GW emits a broadcast message called *end-to-end acknowledgement* (e2e ACK) with a list of acknowledged STAs. Figure 6 shows the e2e ACK operation at the end of every transmission window. Apart from being simple, quick and simultaneously listened by all network elements, e2e ACKs allow STAs to evaluate the state of their path to the GW and act consequently.

The e2e ACK also acts in the HARE protocol stack as an auxiliary mechanism for detecting lost ACKs. Thus, if a child remains awake as a result of not having listened to the ACK of its parent, it will go to sleep when detecting its own ID (and those of its children) in the list of acknowledged STAs from the e2e ACK. Only if at the end of all available transmission windows an STA has not correctly received any acknowledgement (either an ACK or an e2e ACK) will it discard its packet.

#### 5.4.2. Poisoning Mechanism

The poisoning mechanism identifies which specific nodes experience communication problems in their path to the GW, so that they can perform subsequent retransmissions. Nodes having problems with their children transmit packets with the poison flag activated. An STA is considered *poisoned* if, before transmitting an outgoing data packet, one of the following conditions is satisfied:

- The STA is part of a poisoned path; i.e., it has received one or more packets with the poison flag activated during the current transmission window.
- The STA has not received any data packet from one or more of its children.
- The STA has not received all the expected segments from one or more of its children.

**Figure 7:**
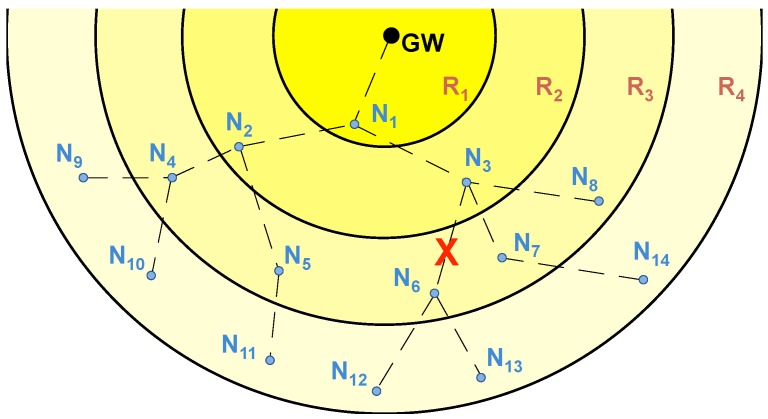
Network topology of the multi-hop LPWAN from Figure 6, with a GW and 14 STAs ($N_{1}$–$N_{14}$) deployed in four rings ($R_{1}$–$R_{4}$).

**Figure 8:**
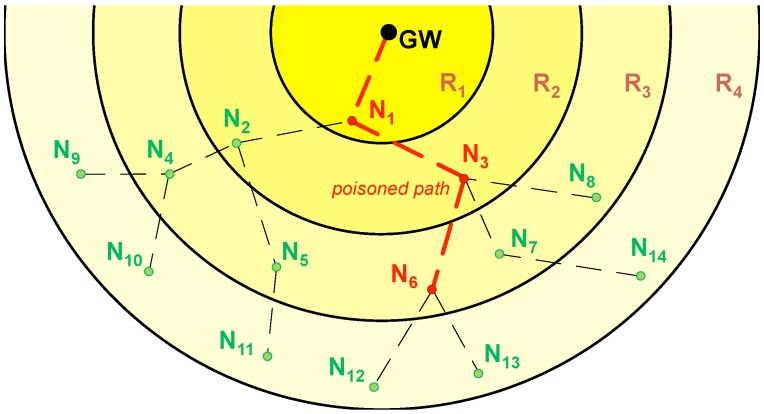
State of the network from Figure 7 after the corresponding e2e ACK. Note the *poisoned path* passing through nodes $N_{6}$, $N_{3}$, and $N_{1}$. Together with the GW, these nodes (colored in red) stay awake during the second transmission window. The rest of nodes (colored in green) go to sleep as they are not involved in the new transmission process.

In Figure 7, node $N_{3}$ activates its poison flag after not receiving data from its child $N_{6}$. In its way to the GW, a data packet from $N_{3}$ poisons its next hop: $N_{1}$. Therefore, nodes $N_{6}$, $N_{3}$, and $N_{1}$ form a *poisoned path*, as shown in Figure 8.

#### 5.4.3. Transmission Windows

A number of transmission windows (w) with their corresponding e2e ACK are included in each uplink data transmission phase to ensure correct packet reception. Within these windows, not all STAs remain awake, but only the ones directly involved in the transmission process. Before the start of a new transmission window, all STAs evaluate whether they shall stay awake or go to sleep.

**Figure 9:**
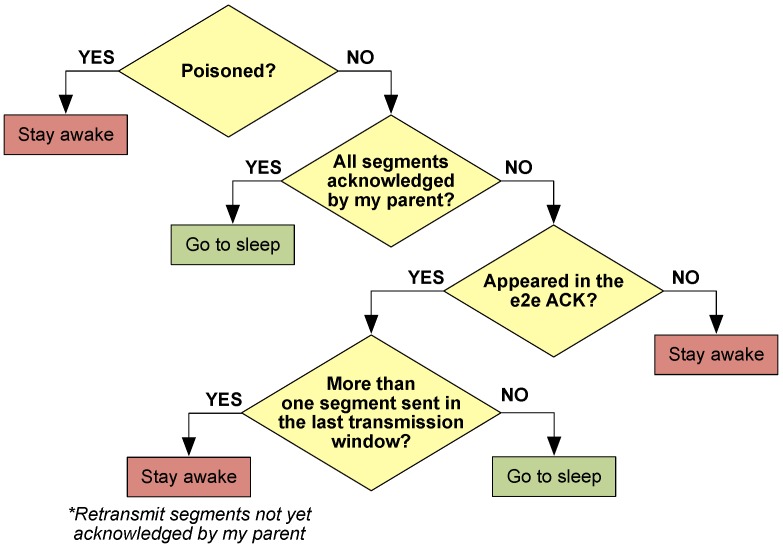
STA’s decision flowchart prior to a new transmission window.

This decision takes into account whether the STA has been previously *poisoned* by one of its children as well as several other restrictions according to the decision flowchart from Figure 9. Whenever an STA decides to go to sleep, it will remain in this state until the next *primary beacon*.

#### 5.4.4. Distributed Caching

Due to the structure of multi-hop networks, lost packets cause expensive retransmissions along every hop of the path between the sender and the receiver [39]. To alleviate this problem, a distributed caching system is used in HARE, so that parents acknowledge the correct reception of packets from children and cache their data until it is properly received in the GW.

As it can be seen in Figure 8, nodes $N_{12}$ and $N_{13}$ can go to sleep after the first transmission window, because their data packets have been acknowledged by node $N_{6}$, which will cache them in memory together with its own data to be sent in the next transmission window.

## 6. Implementation

Contiki 3.0 OS [40] was selected to validate the HARE protocol stack, mainly due to its ability to easily execute multiple processes concurrently and its powerful COOJA network simulator [41]. While COOJA was mainly used in first development stages and early simulations, Zolertia™ RE-Mote nodes [42], whose main features are depicted in Table 5, were used for preliminary testing and operational validation. All results compiled in the current article were obtained by means of Zolertia™ RE-Mote nodes working at 868 MHz. Preliminary testing was also conducted on MEMSIC™ TelosB 2.4 GHz nodes [43], proving that HARE protocol stack is platform-independent. (See [44] for a comprehensive list of hardware compatible with the Contiki OS code tree.).

HARE protocol stack was fully programmed as a new hardware independent module for Contiki 3.0 OS, adding all the described functionalities in Section 5 to the already available upper communication layers (link and network), and including a novel transport layer. Specific interactions of HARE with PHY layers of the aforementioned hardware were programmed separately.

Three different testbeds were designed to test the functionalities of HARE protocol stack running in Zolertia™ RE-Mote nodes: Testbed #1 was used to analyze the performance of main HARE functionalities in a controlled area with easy access to devices, Testbed #2 established the maximum range coverage of the system in line-of-sight condition, and Testbed #3 reproduced the conditions of an environmental monitoring application.

### 6.1. Testbed #1: University Facilities

Performance evaluation was performed in an ad hoc testbed located on the 2nd floor, right wing of the Tanger building at Universitat Pompeu Fabra (UPF) facilities [46]. The testbed consisted of 13 Zolertia™ RE-Mote nodes (one of them acting as a GW and connected to a PC) running the HARE protocol stack with different setups for 15 days in total.

**Figure 10:**
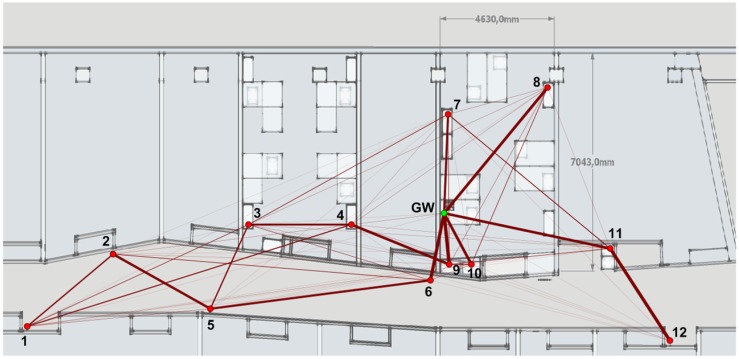
Nodes’ placement and association diagram of Testbed #1 when each STA admits up to five children.

All tests, whose results are compiled in Section 7, were executed considering no mobility and with the same STAs’ placement as in Figure 10. All STAs were powered by an 800 mAh battery except the GW, which was permanently powered by the PC. Results were directly obtained from the GW through the *statistics messages* periodically sent by STAs as well as internal logs. These messages contain information about different metrics such as the number of packets sent and acknowledged, round-trip time (RTT) delays, as well as power profiles of the microprocessor and the radio module.

The calculation of total energy consumption $\left(E_{T}\right)$ is based on two power profiles: $E_{\mu P}$ and $E_{\mathrm{RADIO}}$, for the microprocessor and the radio module, respectively, as shown in Equation (2). $V_{\mathrm{DD}}$ is the supply voltage, while *t* and *I* are, respectively, the time and the current corresponding to the operational states of the microprocessor and the radio module of the employed hardware, whose values are summarized in Table 6. Notice that the $I_{\mathrm{TX}}$ value of the radio module depends on the transmission power level:

(2) $$\begin{array}{c} E_{T}=E_{\mu P}+E_{\mathrm{RADIO}},\\ E_{\mu P}=V_{\mathrm{DD}}\cdot \left(t_{\mathrm{CPU}}\cdot I_{\mathrm{CPU}}+t_{\mathrm{LPM}}\cdot I_{\mathrm{LPM}}\right),\\ E_{\mathrm{RADIO}}=V_{\mathrm{DD}}\cdot \left(t_{\mathrm{RX}}\cdot I_{\mathrm{RX}}+t_{\mathrm{TX}}\cdot I_{\mathrm{TX}}+t_{\mathrm{SL}}\cdot I_{\mathrm{SL}}\right). \end{array}$$

In addition, different network configurations were applied. Firstly, two different RDC sublayers inherent to Contiki OS were tested: NullRDC and X-MAC [48]. While NullRDC maintains STAs continuously awake during *active* periods, X-MAC combines the introduction of sleeping periods for receivers with the use of strobed preambles for senders.

Secondly, and always over the same node deployment, two different network topologies were tested: single-hop and multi-hop. In the first case, all nodes were directly connected to the GW, as in a traditional single-hop LPWAN, while, in the second case, STAs were free to establish their own routes to the GW with the single limitation of having five children per STA.

Thirdly, the whole system was altered with the arbitrarily introduction of a certain error probability when sending both *application packets* and their corresponding ACKs (it is worth noting here that neither messages implied in the association process nor *statistics packets* were affected by arbitrary generated errors to not artificially disturb the network setup nor the collection of operation information). Errors were generated through a uniformly distributed random variable according to mean error values from four different error configurations (see Table 7). Before sending a message, STAs computed this value and discarded messages accordingly.

The addressing system followed the Rime format [49] consisting of two 8-bit numbers. Similarly to IP addressing, the use of netmasks leads to flexible subnetting configurations with up to 2^16^-2 STAs. In our particular case, the first 8-bit number identified the network prefix shared by all devices, and the second one the host part, whose value for GWs was 0 and for STAs was selected from 1 to 255.

All tests began with a *network association primary beacon* in which all nodes tried to associate to the network. From then on, the GW emitted a new (*network association* or *data*) *primary beacon* every $T_{p}=3$ min. *Data primary beacons* could ask STAs for a new *application* or *statistics packet*. In all our tests, *application* and *statistics packets* generated by STAs contained, respectively, $L_{\mathrm{ap}}=10$ bytes and $L_{\mathrm{sp}}=20$ bytes of net information (Implementation of IEEE 802.15.4g in Contiki OS increases the minimum length of any transmitted packet up to 43 bytes after including headers and, if necessary, applying padding.).

### 6.2. Testbed #2: Maximum Range Coverage

With the goal of assessing the suitability of the Zolertia™ RE-Mote as a device for LPWAN applications, a test was performed in the localities of *Prats i Sampsor,* and *Savanastre* (Catalan Pyrenees) to determine its maximum range coverage in line-of-sight condition when using the 868 MHz frequency band. Two Zolertia™ RE-Mote units were used—both of them running the HARE protocol stack: one acting as a GW placed in a static position, and the other as an STA trying to associate to the network from an increasingly greater distance. Selected data rate was r=50 kbps and transmission power was set to its maximum value ($P_{\mathrm{TX}}=14$ dBm).

**Figure 11:**
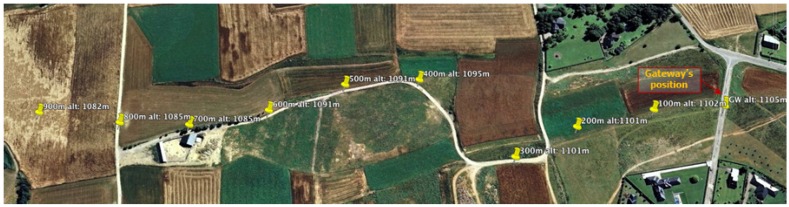
Positions and altitudes of the device acting as STA in Testbed #2.

**Figure 12:**
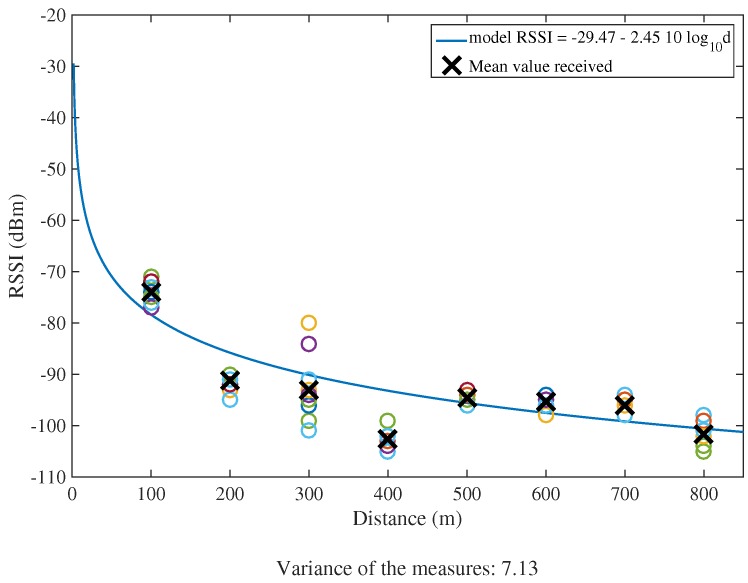
Least square approximation of the simple pathloss propagation model at 868 MHz.

Under these conditions, the maximum distance at which the STA was able to associate to the network created by the GW was ∼850 m. Figure 11 shows the test locations of the STA, and Figure 12 represents the RSSI received at the GW in function of the distance $P_{\mathrm{RX}}\left(d\right)$, according to the propagation model obtained by using the least square approximation method and expressed as:

(3) $$\begin{array}{c} P_{\mathrm{RX}}\left(d\right)=-29.47-2.45\cdot 10\;log_{10}\left(d\right). \end{array}$$

### 6.3. Testbed #3: Environmental Monitoring in Oliveyard

**Figure 13:**
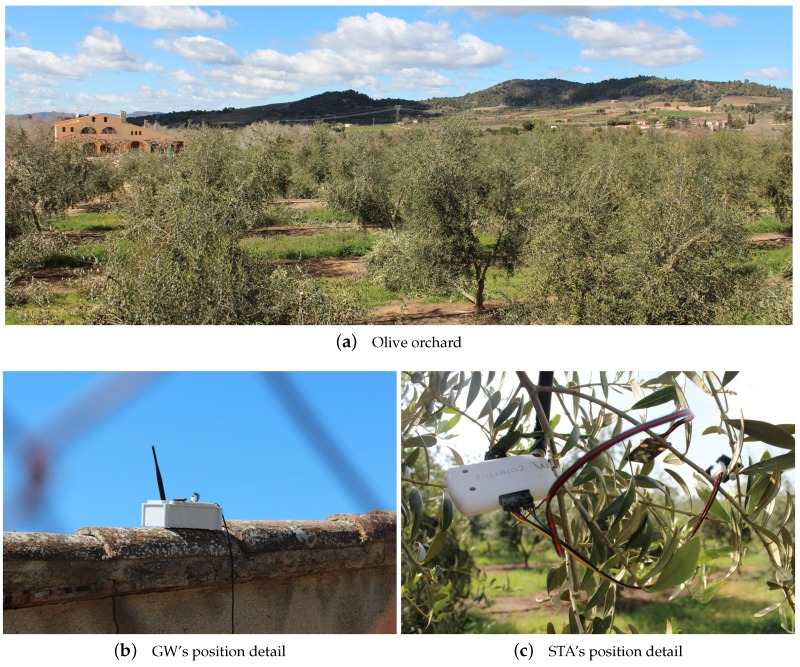
Detail of landscape and devices employed in Testbed #3.

**Figure 14:**
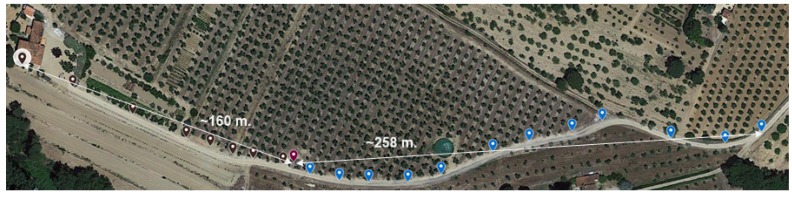
Maximum range coverage achieved at Testbed #3.

On this occasion, the testbed was built in an oliveyard of the *Falset-Marçà Cooperative* [50] from the town of *Falset* (Catalonia), represented in Figure 13a. As in Testbed #2, the first goal was to determine the maximum range coverage of the Zolertia™ RE-Mote nodes in an area where trees may affect the line-of-sight propagation. For this purpose, a GW was placed on the roof of a cabin over 2 m high, as it can be seen in Figure 13b, and an STA was moved further away until it lost the connection with the network. This finally happened at approximately 258 m in east direction and 160 m in west direction from the GW’s position (see Figure 14).

**Figure 15:**
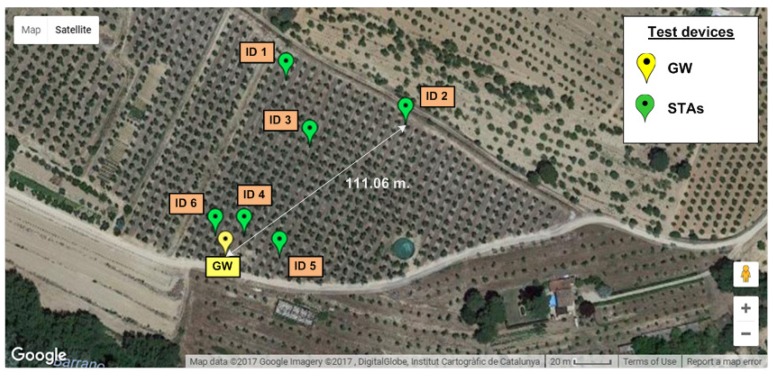
Nodes’ placement at Testbed #3.

Then, 7 Zolertia™ RE-Mote running the HARE protocol stack were deployed on the same oliveyard according to the placement from Figure 15. One of them acted as a GW and was placed again on the roof of the cabin to cover the maximum range, while the rest acted as STAs collecting data from environmental sensors and were placed hanging down from olive tree branches (see Figure 13c). X-MAC was the selected RDC sublayer and the whole system worked under a multi-hop topology limited to five children per STA.

The test lasted four hours, the GW being configured to initially start the beaconing system with a *network association primary beacon* and then ask STAs for new data every 10 min ($T_{p}=10$ min). To send their packets, STAs had five available transmission windows (w=5). Under these conditions, the network self-organized in two rings and achieved a packet delivery ratio (PDR) of 99.26%.

## 7. Results

This section compiles the performance analysis results of HARE protocol stack executed in Testbed #1, whose characteristics are described in Section 6.1.

### 7.1. Association Process

To show the performance and the coherence of the proposed association process and its underlying routing, all STAs were forced to repeatedly renew their association to the network every two *primary beacons* ($N_{\mathrm{pr}}=2$) and compute their best parent according to Equation (1) with $P_{{\mathrm{TX}}_{max}}=14$ dBm and the following empirical parameters: $a_{1}=a_{2}=10$, $a_{3}=1$, and $a_{4}=5$. In turn, GW association parameters were set to values depicted in Table 8.

In addition, the number of children per STA was artificially limited to five to guarantee multiple paths towards the GW. Interspersed *data primary beacons* were used to check the reliability of routing paths and to allow not yet associated STAs to have another opportunity to join the network.

The selected RDC sublayer for all STAs was X-MAC and no error was introduced in the network (i.e., $E_{0/0}$ error configuration was used). Under these premises, and after 200 repetitions, an average number of 11.97 STAs were associated to the network after the *data primary beacon* of the given sequence (i.e., 99.75% of success). As for the PDR, it achieved 100% in all the associated STAs.

Routing tables compiled by the GW were processed and adapted to graphical representation in Figure 10, where line’s thickness is proportional to link’s frequency appearance. Preference of STAs for establishing paths with closer neighbours in their way to the GW becomes evident, just like the importance of *clear paths* (i.e., without obstacles) such as the formed by the corridor walls.

The limitation of five children can be clearly appreciated in STAs #6, #8, #9, #10, and #11 being almost always directly connected to the GW in ring 1. The rest of STAs (principally #7) could only access to that ring when circumstantially having better channel conditions than the aforementioned ones.

### 7.2. Reliability

Once all STAs are associated to the network and their paths to the GW properly established, the next goal is to analyze the reliability and the cost (in terms of energy consumption) of sending data. To do that, the GW was programmed to send 20 beacons with the following sequence: beacon #1 was a *network association primary beacon*, beacons #10 and #20 were *data primary beacons* asking for *statistics packets*; the rest of beacons were *data primary beacons* asking for *application packets*. To send their packets, STAs had five available transmission windows (w=5).

**Figure 16:**
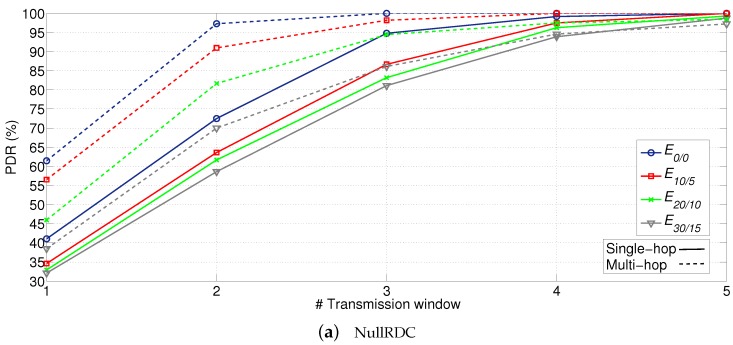

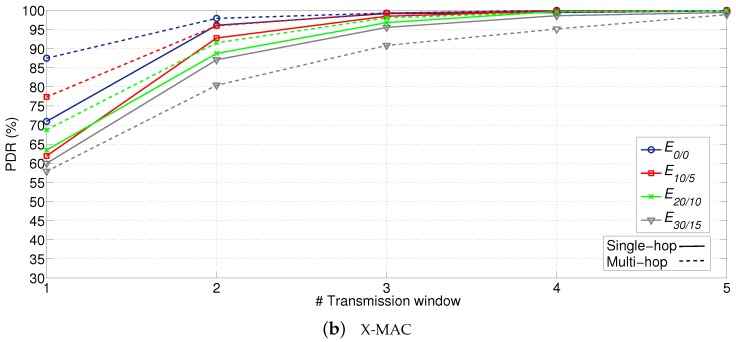
PDR (in %) for different RDC sublayers, network topologies, and error probabilities.

Results with the obtained PDR are compiled in Figure 16. After five transmission windows, PDR is above 95% in any configuration, and it even achieves values above 90% after three and four transmission windows when using X-MAC and NullRDC, respectively. In this case, NullRDC specially suffers from the effect of collisions, due to the CSMA/CA backoff implementation and the higher number of concurrently active STAs compared to X-MAC. (Main values of the CSMA/CA default backoff implementation in Contiki OS: minimum value of the backoff exponent (macMinBE=0), maximum value of the backoff exponent (macMaxBE=4), and maximum number of backoff attempts (macMaxCSMABackoffs=5).).

Another insight from obtained results is how multi-hop topology outperforms single-hop in all possible configurations except when using X-MAC with $E_{30/15}$ and, in an almost imperceptible extent, in transmission window #5 of extremely high error-prone cases. Again, the inherent reduction of concurrently active STAs competing for the channel during the same time period (in this case, due to the allocation of STAs to different slots according to their ring) proves beneficial for system’s reliability.

**Figure 17:**
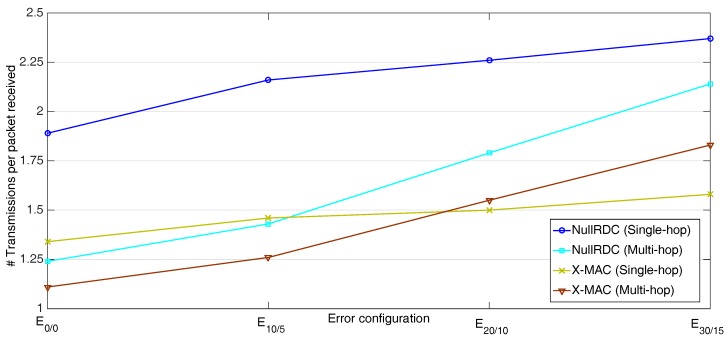
Number of transmissions per packet received.

The network’s ability to properly deliver data packets to its destination was also analyzed by computing the quotient between the total number of packets sent by STAs and those properly received by the GW. As shown in Figure 17, multi-hop schemes have better performance than single-hop except when significant high-error rates are applied. In highly unfavorable channels, parents usually do not receive all their expected payloads at once, so that they tend to send several packets in successive transmission windows with only partial information.

### 7.3. Energy Consumption

**Figure 18:**
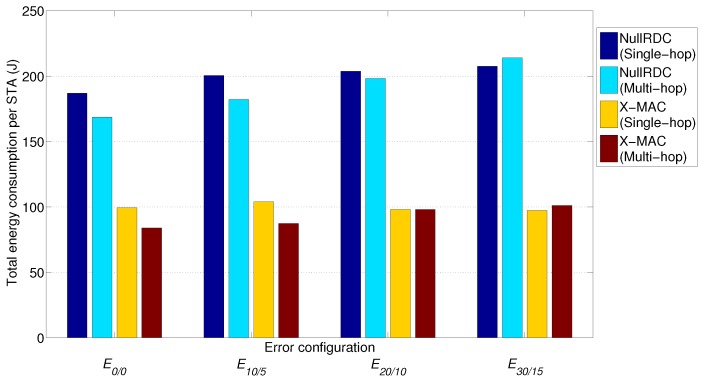
Average total energy consumption per STA after a 1-hour test with $T_{p}=3$ min.

The effect of this interdependence can also be observed in total energy consumption (Figure 18), computed after 20 transmitted beacons (i.e., a 1-h test). Important savings (up to 15%) can be achieved when using multi-hop schemes with respect to single-hop ones in not extremely high-error configurations $(E_{0/0}$–$E_{20/10})$ and similar or slightly worse values (less than 4% of extra consumption) in $E_{30/15}$. From previous studies [38], we believe that in larger networks, these gains will be much higher.

Time percentage of STAs’ microprocessors in low power mode is, in all studied cases, above 97% for X-MAC and 99% for NullRDC, due to the higher number of operations involved in the first case. However, the impact of radio module sleeping periods introduced by X-MAC reduces total energy consumption in up to 50% with respect to NullRDC. Consequently, values of energy consumed per bit of payload delivered are confined between 50–65 mJ/bit for X-MAC, and 105–140 mJ/bit for NullRDC.

As for the battery lifetime, Table 9 compiles the duration in days of the 800 mAh battery included in the Zolertia™ RE-Mote with $T_{p}=3$ min, as well as two estimations with $T_{p}=1$ h and $T_{p}=4$ h. The temporal flexibility of the TDMA-based system employed in HARE allows this kind of extrapolations, by assuming that, in non-active time periods, both the microprocessor and the radio module remain asleep.

### 7.4. Delay

The time difference between the transmission of a data packet and the proper reception of the e2e ACK determines the end-to-end communication delay of an STA ($D_{e2e}$). This value can be computed by using the following equation:

(4) $$\begin{array}{c} D_{e2e}=T_{r}+\left(r-1\right)\cdot T_{r}+\left(i-1\right)\cdot R\cdot T_{r}=\left(r+(i-1)\cdot R\right)\cdot T_{r}, \end{array}$$

where $T_{r}$ is the duration of a ring slot, *r* is the ring to which the STA belongs, *i* is the index of the transmission window in which the STA receives the e2e ACK (with i∈[1,w]), and *R* is the total number of network rings.

**Figure 19:**
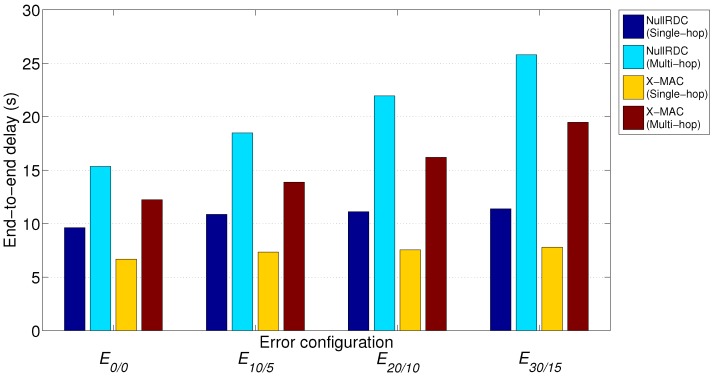
Average end-to-end delay when $T_{r}=5$ s.

Figure 19 shows the computed average end-to-end communication delay ($\bar{D_{e2e}}$) of uplink transmissions from Section 7.2 for the two considered RDC sublayers and the two different network topologies. While the ring slot duration has a constant value of $T_{r}=5$ s, the rest of variables (*r*, *i*, and *R*) varied depending on the network setting, and were obtained from *statistics packets* gathered by the GW.

As expected, multi-hop topologies suffer from higher delays (always at least 50% more than single-hop links in the considered cases) as messages must go through several hops until reaching their destination. With regard to RDC sublayers, X-MAC outperforms NullRDC in terms of delay, as STAs can send their messages *earlier* (i.e., without needing so many transmission windows).

Although end-to-end delay is a clear disadvantage of multi-hop topologies, the HARE protocol stack is intended to be used in non-delay sensitive applications. In any case, these values could be improved by adjusting the number of transmission windows (*w*), the duration of each ring slot ($T_{r}$), or simply by developing schemes where STAs sent critical and/or event-based traffic (i.e., alarms) directly to the GW by using a reserved time slot.

### 7.5. Throughput

Prior to its analysis in the performed tests, a theoretical computation of the maximum expected throughput ($T_{\max}$) for the network configuration of Section 7.2 is provided below. Firstly, time between two consecutive *primary beacons* is forced to be minimum ($T_{p_{min}}$), as a result of not considering secondary beacons (i.e., $k_{s}=0$) and only summing the time for the *STA association mechanism* and that reserved to data transmissions:

(5) $$\begin{array}{c} T_{p_{min}}=\left(a_{s}\cdot T_{a}+T_{g}\right)\cdot a_{t}+w\cdot R\cdot T_{r}. \end{array}$$

Then, considering that all network STAs ($N_{\mathrm{STA}}$) follow the same beacon scheduling from Section 7.2 (i.e., 1 *statistics packet* every nine consecutive *application packets*) and all transmitted packets are received by the GW, $T_{\max}$ is obtained as:

(6) $$\begin{array}{c} T_{\max}=\frac{N_{\mathrm{STA}}\cdot \left[\left(\frac{9}{10}\right)\cdot L_{\mathrm{ap}}+\left(\frac{1}{10}\right)\cdot L_{\mathrm{sp}}\right]}{T_{p_{min}}}=\frac{N_{\mathrm{STA}}\cdot \left[\left(\frac{9}{10}\right)\cdot L_{\mathrm{ap}}+\left(\frac{1}{10}\right)\cdot L_{\mathrm{sp}}\right]}{\left(a_{s}\cdot T_{a}+T_{g}\right)\cdot a_{t}+w\cdot R\cdot T_{r}}, \end{array}$$

where $L_{\mathrm{ap}}$ and $L_{\mathrm{sp}}$ are, respectively, the size of the net information contained in an *application packet* and in a *statistics packet*. Lastly, assuming $N_{\mathrm{STA}}=12$, $L_{\mathrm{ap}}=10$ bytes, $L_{\mathrm{sp}}=20$ bytes, and setting GW association parameters to those of the *STA association mechanism* from Table 8, computed $T_{p_{min}}$ and $T_{\max}$ for different *R* values are compiled in Table 10.

The highest throughput value $T_{\max}=25.76$ bits/s corresponds to the single-hop case (R=1), when time between *primary beacons* is $T_{p_{min}}=41$ s. For higher *R* values, the growth of $T_{p_{min}}$ is inversely proportional to the decrease of $T_{\max}$, with expected values below 10 bits/s.

**Figure 20:**
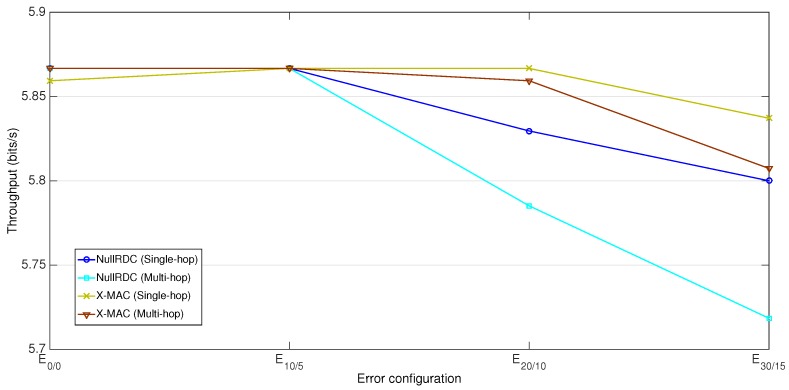
Computed throughput for the different error configurations.

Figure 20 reflects the measured throughput in the different network configurations of Section 7.2, where $T_{p}$ was set to 3 min. With a throughput of 5.87 bits/s, this value is far from those of Table 10. In our experiments, the use of a more conservative $T_{p}$ value, which deliberately leaves the channel empty for certain time periods, results in less use of available resources.

While both employed RDC sublayers behave almost equally (achieving their maximum throughput values in low error-prone configurations), X-MAC responds better than NullRDC in $E_{20/10}$ and $E_{30/15}$ due to its lesser exposure to packet losses. As for network topologies, and again being consistent with reliability results, single-hop cases perform slightly better than multi-hop ones in high error-prone scenarios.

### 7.6. Resilience against Failures

To prove the adaptability and resilience of the routing protocol implemented in HARE, the network was subjected to the deliberate shutdown of two of its STAs. With respect to the GW, it was programmed to send 50 beacons with the following sequence: beacon #1 was a *network association primary beacon*, beacons multiple of 10 were *data primary beacons* asking for *statistics packets*; the rest of beacons were *data primary beacons* asking for *application packets*. In addition, the *disassociation mechanism* was programmed in the GW to remove an STA from the network if not receiving any of its data packets during one *primary beacon* ($N_{\mathrm{pd}}=1$).

**Figure 21:**
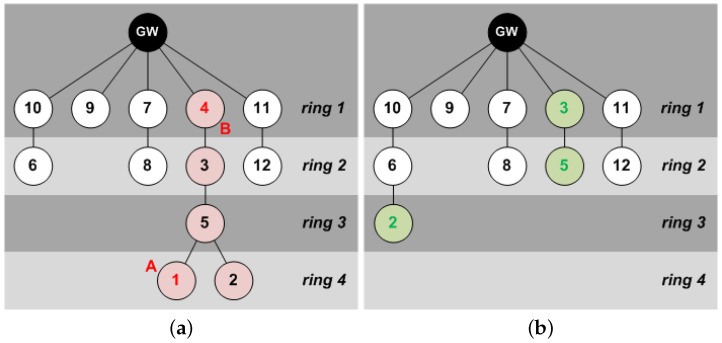
Network topology before and after shutdown of nodes #1 and #4. (**a**) logical network topology after the *network association primary beacon*; (**b**) logical network topology from beacon #15 until beacon #50.

Once the initial *network association mechanism* is finished, the network was organized in four rings, as shown in Figure 21a. After beacon #4 (**A**), STA #1 was switched off, but it did not imply further problems to the network, as this STA did not have any children. However, after beacon #12 (**B**), STA #4 was also switched off, and it forced the network to reconfigure itself. The path to the GW of STAs #2, #3 and #5 was broken, and they had to look for a new route by using the *STA association mechanism* of successive *data primary beacons*. After beacon #15, all active STAs (i.e., all of them except #1 and #4, which remained off) had a path to the GW and the network was stable again (see Figure 21b).

### 7.7. Power Regulation Mechanism Performance

The previous test was also useful to analyze the performance of the PRM when setting it with ${\mathrm{RSSI}}_{min}=-110$ dBm and ${\mathrm{RSSI}}_{max}=-100$ dBm. It is worth noting here that Zolertia™ RE-Mote devices use up to 31 different power levels (from −16 dBm to 14 dBm with steps of 1 dB [51]) and are programmed by default with the maximum transmission power level.

**Figure 22:**
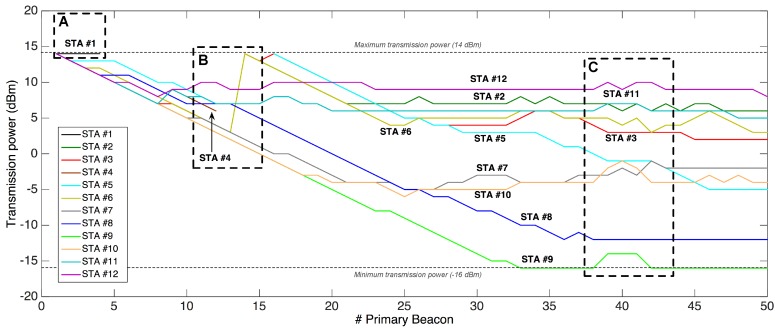
Temporal evolution of STAs’ transmission power level in multi-hop setting.

Figure 22 shows the clear reduction of transmission power in most of the analyzed STAs during 50 *primary beacons*, the most significant examples being STAs #7, #8, #9 and #10; that is, the nearest ones to the GW. This fact results in a lower energy consumption, as $I_{TX}=61$ mA when transmitting at 14 dBm, but almost half ($I_{TX}=39$ mA) when doing it at –16 dBm.

The effects of switching off nodes are also visible in the transmission power, as shown in (**A**) and (**B**) from Figure 22. While STA #1 in (**A**) simply stopped working, nodes involved in the shutdown of STA #4 in (**B**) experienced notable changes. Thus, STAs #2, #3 and #5 *disappeared* along with the shutdown of STA #4. However, they became associated again between beacons #13 and #15 with maximum transmission power. For its part, when STA #6 became a parent of STA #2, it set the maximum power level to establish connection with its new child.

The PRM also proved its good performance against channel alterations as shown in area (**C**). In this case, and due to the test execution on a real scenario, the presence of people in the floor corridor may have disturbed channel conditions. To overcome this situation, some STAs (#9, #10, #11, and #12) selected greater transmission power levels temporarily, which were reestablished once the detected channel issues were finished.

**Figure 23:**
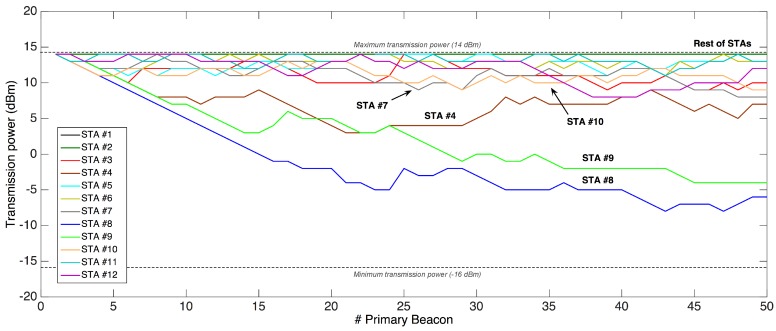
Temporal evolution of STAs’ transmission power level in a single-hop setting.

To evaluate the effectiveness of the PRM in single-hop networks, this test was repeated with the same configuration except from the network topology, which was forced to be single-hop. As it can be seen in Figure 23, only STAs located very close to the GW and/or with favorable channel propagation conditions (that is, STAs #4, #8, and #9) managed to noticeably reduce their transmission power over the test’s duration. As for the rest of STAs, they could not decrease significantly their power level, and tended to stabilize them from half of the test.

Lastly, it is relevant to note the behavior of STA #9, whose final power level was well over that of the multi-hop test, when that STA belonged to the first network ring. This difference is due to the inherent operation of the PRM, which increases in one unit the power level in each successive retransmission, and is consistent with reliability results from Section 7.2, where measured PDR in first transmission windows is always higher in multi-hop approaches.

## 8. Conclusions

Single-hop has become the de facto topology to transmit data in current LPWANs mainly due to the need for network simplicity and robustness, and the fear of consuming too much energy in processing tasks and/or maintaining complex routing mechanisms. However, the HARE protocol stack presented in the current article proves the suitability of alternative uplink multi-hop communication approaches. Distributed among three OSI layers (link, network and transport), the multiple mechanisms contained in HARE ensure network reliability and resilience against failures in uplink transmissions while keeping low energy consumption.

Results from a real testbed show uplink PDR values above 95% for all considered configurations, with faster achievement of this level when using multi-hop topologies with multiple transmission windows. In addition, multi-hop topologies outperform single-hop ones in terms of energy consumption in the considered non error-prone scenarios, with up to 15% improvement (which could even be much higher in larger networks) and values as low as 50 mJ/bit when employing an effective RDC sublayer. Similarly, network auto-configuration and resilience have been successfully put to the test after forcing the shutdown of some network STAs.

In the near future, LPWANs are foreseen to occupy a central role in applications requiring to interconnect low-bandwidth devices, focusing on range and power efficiency. While range coverage is mostly an issue from the physical layer, future challenges regarding power efficiency will surely encompass the coordination of different layers and even the inclusion of novel cross-layer mechanisms.

## Figures and Tables

**Table 1:** Common requirements of HARE use cases.

Requirement	Value
Coverage range	Up to several km
Geographic coverage	Excellent even in remote and rural areas
Penetration	Good in-building and in-ground penetration
Device density (per base station)	High (up to thousand)
Power profile	Unassisted, battery-powered devices
Battery lifetime	From some months up to several years
Throughput	<100 bits/s
Latency	Non-delay sensitive
Mobility	Static devices
Cost	Low hardware and operating cost
Maintenance	Unassisted and self-organizing network
Delivery model	Continuous data delivery model

**Table 2:** Use cases supported by HARE protocol stack.

Sector	Use Cases
**Home automation**	Domotics
	Child/elderly tracking
	Smart metering
**Industrial automation**	Remote maintenance/control
	Distribution automation (smart grid)
	Logistics
	Local asset tracking management
	Smart metering
**Public infrastructure**	City smart lighting
	Smart parking
	Intelligent buildings
	Predictive maintenance
**Natural resources**	Environmental monitoring
	Natural disasters detection
**Smart agriculture and farming**	Agriculture monitoring
	Animal monitoring
	Silo stock monitoring

**Table 3:** Main features of HARE protocol stack.

Layer	Features
**Transport**	End-to-end acknowledgement (e2e ACK) Poisoning mechanism (packet loss detection) Transmission windows Distributed caching
**Network**	Addressing system Association Routing
**Link**	Beaconing system Wakeup patterns Data transmission, aggregation and segmentation Power regulation mechanism
**Physical**	*Hardware dependant*

**Table 4:** HARE link layer.

MAC Sublayer		RDC Sublayer
Scheduling-Based Protocol	Contention-Based Protocol	
TDMA	CSMA/CA, *or other*	NullRDC, X-MAC, *or other*

**Table 5:** Main features of Zolertia™ RE-Mote nodes.

	Zolertia™RE-Mote
Microprocessor	ARM Cortex-M3
Radio Module	TI CC1200 [45]
Frequency Band	868/915 MHz
Modulation	2-GFSK
Data rate	50 kbps
Underlying communication standard	IEEE 802.15.4 g

**Table 6:** Current values of Zolertia™ RE-Mote operational states (from [47]).

	Operational State	Current
**Microprocessor** ARM Cortex-M3	Processing (CPU)	$I_{\mathrm{CPU}}=13$ mA
	Low power mode (LPM)	$I_{\mathrm{LPM}}=0.4$μA
**Radio Module** TI CC1200	Receiving (RX)	$I_{\mathrm{RX}}$ = 19 mA
	Transmitting (TX)	$I_{\mathrm{TX}}$ = 39–61 mA
	Sleeping (SL)	$I_{\mathrm{SL}}$ = 0.12 μA

**Table 7:** Definition of error configurations in the proposed testbed.

Error Config.	Data Error	ACK Error
$E_{0/0}$	0%	0%
$E_{10/5}$	10%	5%
$E_{20/10}$	20%	10%
$E_{30/15}$	30%	15%

**Table 8:** GW association parameters.

	$a_{t}$	$a_{s}$	$T_{a}$	$T_{g}$
*Network association mechanism*	5	6	2 s	8 s
*STA association mechanism*	1	4	2 s	8 s

**Table 9:** Average lifetime of an 800 mAh battery.

			Battery Lifetime (Days)		
			$T_{p}=3$ min	$T_{p}=1$ h	$T_{p}=4$ h
**NullRDC**	**Single-hop**	$E_{0/0}$	2.37	47.35	187.87
		$E_{10/5}$	2.21	44.19	175.41
		$E_{20/10}$	2.18	43.46	172.53
		$E_{30/15}$	2.14	42.70	169.53
	**Multi-hop**	$E_{0/0}$	2.63	52.51	208.17
		$E_{10/5}$	2.44	48.60	192.79
		$E_{20/10}$	2.24	44.66	177.27
		$E_{30/15}$	2.07	41.35	164.23
**X-MAC**	**Single-hop**	$E_{0/0}$	4.46	88.75	349.64
		$E_{10/5}$	4.27	85.00	335.07
		$E_{20/10}$	4.52	89.99	354.46
		$E_{30/15}$	4.56	90.79	357.55
	**Multi-hop**	$E_{0/0}$	5.29	105.17	413.16
		$E_{10/5}$	5.08	101.04	397.21
		$E_{20/10}$	4.53	90.10	354.85
		$E_{30/15}$	4.39	87.38	344.31

**Table 10:** $T_{p_{min}}$ and $T_{\max}$ values in function of *R*.

Number of Rings	$T_{p_{\min}}$ (s)	$T_{\max}$ (bits/s)
R=1	41	25.76
R=2	66	16
R=3	91	11.60
R=4	116	9.10
R=5	141	7.49
